# Supplementary material for: Methods for improving participation rates in national self-administered web/mail surveys: Evidence from the United States
Source: PLoS One. 2023 Aug 4;18(8):e0289695. doi: 10.1371/journal.pone.0289695 (PMC10403122; doi:10.1371/journal.pone.0289695)
Supplement: S1 Appendix — (PDF) [file pone.0289695.s001.pdf]

Supporting Information for "Methods for Improving Participation Rates in National Self-Administered Web/Mail Surveys: Evidence from the United States"

Appendix I: Esri Tapestry Segmentation

Esri Tapestry Segmentation is a geographic market segmentation system that uses cluster analysis to divide block groups across the United States into 14 LifeMode groups. Each LifeMode group represents a type of neighborhood that has distinctive features based on data from 2010 Census, the American Community Survey, Esri's demographic updates and consumer surveys such as the Survey of the American Consumer (Esri, 2021). The LifeMode groups are briefly summarized in Table S1, ordered by income. Please refer to the Esri website for detailed descriptions: https://doc.arcgis.com/en/esri-demographics/data/tapestry-segmentation.htm

Some LifeMode groups have features that are related to how comfortable the residents may be with the Internet, which in turn might affect their web/PAPI preferences. In general, the Internet use is estimated to be more prevalent among the younger, higher-income, higher-educated, and urban/suburban populations (Pew Internet/Broadband Fact Sheet, 2021). We bold the related features in Table S1.

Table S1. The 14 LifeMode groups of the Esri Tapestry Segmentation system. Features related to web/PAPI preferences are highlighted in bold.

LifeMode	Definition
Affluent Estates	Established wealth<br>**Well-educated** and well-traveled married couples<br>90% homeowners<br>Participate actively in communities
Upscale Avenues	Prosperous married couples living in older suburban enclaves

	70% homeowners; financially responsible
Uptown Individuals	**Young successful singles** in the city<br>The **highest educated**<br>**Internet dependent**
Family Landscapes	Successful two-worker young families in their first homes<br>In suburban or semirural areas with a low vacancy rate
GenXurban	Middle-aged Generation X married couples and a growing population of retirees<br>Second-largest Tapestry group
Cozy Country Living	Empty nesters in **bucolic** settings<br>Largest Tapestry group; half of households locate in the Midwest<br>**Spend little time online**
Ethnic Enclave[1]	Young, Hispanic homeowners with families<br>Multilingual and multigenerational households with 2nd/3rd/4th generation Hispanic children
Middle Ground	Lifestyles of thirtysomethings (Millennials)<br>Majority of residents attended college or attained a college degree<br>**Online all the time**
Senior Styles	Saving for **retirement**<br>Married empty nesters or singles living alone<br>Cellphone + landlines; **some prefer print media**
Rustic Outposts	**Country life** with **older families** in older homes<br>Pay bills **in person**; use the yellow pages; read newspapers, magazines, and mail-order books
Midtown Singles	Millennials on the move – single and urban<br>Working in service and unskilled positions<br>**Embrace the Internet**
Hometown	Growing up and staying close to home<br>Close-knit urban communities of young singles (many with children)<br>Religion is the cornerstone
Next Wave	Urban dwellers; young, hardwarding families<br>A large share are foreign born and speak only their native language (mostly Hispanic)
Scholars and Patriots	College and military populations; the **youngest** group

Highly mobile renters
Tethered to their phones and electronic devices

[1] Ethnic Enclave has been renamed to Sprouting Explorers recently. We continue using the old name because the LifeMode was named Ethnic Enclave when we obtained the data.

In the analyses reported in the main text, we compared screener response rates across Esri LifeMode groups. The interpretation of the differential response rates across LifeModes can be facilitated by understanding the associations between LifeModes and key outcome variables. If LifeModes have no associations with the outcome variables, then the differential response rates will likely not lead to biases in survey estimates. On the other hand, if the LifeModes are associated with key outcome variables, then the differential response rates increase the risk of nonresponse biases. Thus, we analyzed the associations between LifeModes with a series of key outcome variables in the AFHS. Specifically, we are interested in knowing whether LifeModes can stand out as a meaningful predictor of outcome variables in addition to other auxiliary variables.

Many auxiliary variables can be linked to the AFHS sample, including those from the sampling frame, commercial sources, and large datasets on the block groups (from the Decennial Census and American Community Survey) and census tracts (National Neighborhood Data Archive, NaNDA: https://www.openicpsr.org/openicpsr/nanda). However, for predicting outcome variables, the information contained in these individual variables tends to be thin. We consider Esri's LifeModes as a summary of many of the auxiliary variables, because LifeModes are the results of cluster analysis in which Census and ACS variables are the major source of input data. Our analysis follows the following steps:

1. We compiled a list of 97 auxiliary variables from 1) the sample frame (e.g., Census division), 2) the screener interview (e.g., age of sampled individuals), 3) Census and ACS variables at the block group level (e.g., percentage of the foreign-born population in block groups), 4) NaNDA variables at the census tract level (e.g., the proportion of land with high-intensity development), and 5) Esri LifeMode groups.
2. We identified 34 key outcome variables from AFHS (e.g., ever smoked 100 cigarettes). All outcome variables were coded into binary variables.
3. For each key outcome variable, we fitted LASSO logistic regression models using main effects of all auxiliary variables with 10-fold cross-validation. We identified variables that consistently emerged as important predictors across the validation folds. Then we defined a union of the important predictors across all outcome variables.

This process identified 74 important predictors for AFHS key outcome variables. Since many of them are dummy variables, they correspond to 41 auxiliary variables. It is noteworthy that Esri's LifeModes consistently emerged as important predictors across many outcome variables. In fact, all 13 dummy variables defined by the LifeModes were identified as important predictors. Such results suggest that the LifeMode groups are associated with the AFHS outcome variables. The effects of the LifeMode groups remain noticeable even when many other auxiliary variables are included in the models. The associations between LifeModes and the outcome variables reinforce the value of investigating response rates across the LifeModes.

Appendix II: Response Rate Calculations

This section of the supporting appendix explains how the response rates reported in the main text were calculated.

Screening Response Rate

The main text reports that the response rate of the screening phase was 15.0%. The screening response rate was calculated as

$$\frac{Elig + Inelig}{All - NS - (UH)(1 - e2)}$$

where:

Elig=Screened eligible

Inelig=Screened ineligible

All=All released sample

NS=Screened non-sample, including mail returned as vacant or no such number or no such street.

UH=Unknown household

e_2= 88.94%, the estimated occupancy rates of the UH cases. The estimation of e_2 is explained below.

The counts of AFHS replicate 1 sample units in the AAPOR response rate categories are summarized in the table below.

ScreenOutcome	AAPORCategory	Known	Frequency
ScreenNS	NS	Non-HH	668

ScreenOther_Final	UH		15137
ScreenOther_Final	UO	HH	1020
ScreenedEligible	I	HH	801
ScreenedEligible	O	HH	435
ScreenedEligible	P	HH	197
ScreenedEligible	R	HH	80
ScreenedIneligible	Inelig	HH	1043
Total			19381

Among the cases which household status was *known*, the occupancy rate was

$$e_1 = \frac{1020 + 801 + 435 + 197 + 80 + 1043}{1020 + 801 + 435 + 197 + 80 + 1043 + 668} = 84.26\%$$

This observed occupancy rate is different from the 87.91% estimated occupancy rate based on ACS 2019 one-year data. We assume that this difference comes from the 15,137 cases in which household status was unknown (UH). By matching the AFHS full sample occupancy rate to the ACS occupancy

$$\frac{1020 + 801 + 435 + 197 + 80 + 1043 + 15137 * e_2}{19381} = 87.91\%,$$

we solved that the estimated occupancy rate of the UH cases e_2 is 88.94%.

Main Response Rates

The main text reports that the response rate of the main phase was 66.0%, conditional on participation in the screening phase. The main response rates were calculated as

$$\frac{I + P}{Elig}$$

where:

I=Interview

P=Partial

Elig=Screened Eligible

Overall Response Rates (RR4)

The main text reports that the net AAPOR RR4 response rate[1] was 9.9%. This is obtained by the product of screening response rate and main response rate.

[1] The American Association for Public Opinion Research (AAPOR), *the Standard Definitions - Final Dispositions of Case Codes and Outcome Rates for Surveys*. https://www.aapor.org/AAPOR_Main/media/publications/Standard Definitions20169theditionfinal.pdf

Appendix III: Online Consent Form

The American Family Health Study

Online Survey Interview Consent Form for Modules 1-3 or Full Instrument

You are invited to take part in *The American Family Health Study*. This study is aimed at assessing the health of American individuals and families. The survey includes questions about health, family life, and other related topics. The information you provide will be used for scientific research designed to help improve health services and health education programs. Completing this survey will take around [15/45] minutes. Once this survey is complete we will send a [$20/$30/$70] check in appreciation of your time. Your participation is voluntary.

The knowledge gained from this study is important for understanding how common experiences in life affect health. Your participation will give researchers a better understanding of the factors that influence health such as health care, marriage and divorce, having and raising children, and other life experiences. This information may be used to create policies and programs that help people in the future. Your participation is important because it will help the study accurately represent people like you.

For this survey, you will be asked questions online about your personal life. Answering these questions will take about [15/45] minutes. This survey will ask questions related to relationships, fertility, family dynamics, education, employment, doctor visits, health insurance, and related attitudes and behavior. Some questions ask about potentially private, personal matters such as your experiences with sexual health. You may choose not to answer any question for any reason. Participants will be asked to take [one survey/a total of three surveys about two months apart]. The data collected will inform policies and programs related to the health and well-being of American individuals and families. We are grateful for your participation.

We believe there are very few risks involved in participating in this study. It is possible that you may feel discomfort by being asked personal questions. You may skip answering any of the questions. There may be unforeseen privacy risks, but we believe that the risks are very small. We follow stringent practices to maintain the security of all data that could identify you. You may not receive any personal benefits from being in this study. However, others may benefit from the knowledge gained from this study. Your participation is voluntary, and you may leave the study at any time. If you leave the study before it is finished, there will be no penalty to you. You will receive a [$20/$30/$70] check once you have completed the survey and submitted your responses online.

Please choose a private place to complete your survey. Your answers to the questions will be kept confidential. Your answers will be used for research only and will be stored in secure data repositories for future research studies without your additional informed consent. Researchers accessing the data will need to describe how they will use the data and pledge data confidentiality. These researchers will never have access to any information that will personally identify you as a respondent. Only coded information that has been stripped of direct identifiers will be stored in the repositories. The link between study codes and direct identifiers will be kept securely by the study and will never be made public. There is no limit on the length of time we will store your information.

A Department of Health and Human Services Certificate of Confidentiality covers this research in order to ensure your privacy. This means that we cannot be forced to disclose any research

information that may identify you, even by a court subpoena, in any federal, state, or local civil, criminal, administrative, legislative, or other proceedings.

The Certificate of Confidentiality does not prevent you or a member of your family from voluntarily releasing information about yourself or your involvement in this research. If an insurer, employer, or other person obtains your written consent to receive research information, then we will not use the Certificate to withhold that information. The Certificate cannot be used to resist a demand for information from the United States Government that is solely used for auditing or evaluation of federally funded projects. We may report to state or local officials evidence of harm or abuse to any vulnerable person, but we will not ask you any questions about such topics.

We would be more than happy to answer any questions you have about the study. Please call the University of Michigan Survey Research Center, toll free, at **1-8xx-xxx-xxxx** or email the American Family Health Study at **afhs-info@umich.edu**. The AFHS website also has interesting information about the study — including about how the data will be used to inform American public health policies and programs — at http://afhs.isr.umich.edu.

If you have questions about your rights as a research participant, or wish to obtain information, ask questions or discuss any concerns about this study with someone other than the researcher(s), please contact the University of Michigan Health Sciences and Behavioral Sciences Institutional Review Board, 2800 Plymouth Road, Building 520, Room 1169 Ann Arbor, MI 48109-2800, (734) 936-0933 [or toll free, (866) 936-0933], irbhsbs@umich.edu. Please refer to protocol HUM00167171.

The Principal Investigator leading this study is Dr. Brady T. West of the University of Michigan. This study is supported by the Eunice Kennedy Shriver National Institute of Child Health & Human Development of the National Institutes of Health under Award Number R01HD095920. The content is solely the responsibility of the authors and does not necessarily represent the official views of the National Institutes of Health.

By clicking yes below, you are agreeing to participate in the study. You may print this page for your records. You may also request a copy of this page by calling **1-8xx-xxx-xxxx** or emailing us at **afhs-info@umich.edu**.

Yes, I agree with the terms above.	No, I do not agree with the terms above.

Appendix IV: Mailed Consent Form

The American Family Health Study

Mail Survey Interview Consent Form for Modules 1-3 or Full Instrument

You are invited to take part in *The American Family Health Study*. This study is aimed at assessing the health of American individuals and families. The survey includes questions about health, family life, and other related topics. The information you provide will be used for scientific research designed to help improve health services and health education programs. Completing this survey will take around [15/45] minutes. Once this survey is complete we will send a [$20/$30/$70] check in appreciation of your time. Your participation is voluntary.

The knowledge gained from this study is important for understanding how common experiences in life affect health. Your participation will give researchers a better understanding of the factors that influence health such as health care, marriage and divorce, having and raising children, and other life experiences. This information may be used to create policies and programs that help people in the future. Your participation is important because it will help the study accurately represent people like you.

For this survey, you will be asked questions about your personal life. Answering these questions will take about [15/45] minutes. This survey will ask questions related to relationships, fertility, family dynamics, education, employment, doctor visits, health insurance, and related attitudes and behavior. Some questions ask about potentially private, personal matters such as your experiences with sexual health. You may choose not to answer any question for any reason. Participants will be asked to take [one survey/a total of three surveys about two months apart]. The data collected will inform policies and programs related to the health and well-being of American individuals and families. We are grateful for your participation.

We believe there are very few risks involved in participating in this study. It is possible that you may feel discomfort by being asked personal questions. You may skip answering any of the questions. There may be unforeseen privacy risks, but we believe that the risks are very small. We follow stringent practices to maintain the security of all data that could identify you. You may not receive any personal benefits from being in this study. However, others may benefit from the knowledge gained from this study. Your participation is voluntary, and you may leave the study at any time. If you leave the study before it is finished, there will be no penalty to you. You will receive a [$20/$30/$70] check once we receive your completed survey questionnaire in the mail.

Please choose a private place to complete your survey. Your answers to the questions will be kept confidential. Your answers will be used for research only and will be stored in secure data repositories for future research studies without your additional informed consent. Researchers accessing the data will need to describe how they will use the data and pledge data confidentiality. These researchers will never have access to any information that will personally identify you as a respondent. Only coded information that has been stripped of direct identifiers will be stored in the repositories. The link between study codes and direct identifiers will be kept securely by the study and will never be made public. There is no limit on the length of time we will store your information.

Immediately place your completed survey in the return envelope provided and seal the envelope. Place the envelope in any USPS mailbox, or place the envelope in your personal

mailbox to be picked up by your mail carrier. (No stamps required - the postage is already paid for by the study.)

By returning the survey questionnaire enclosed, you are agreeing to participate in the study.

A Department of Health and Human Services Certificate of Confidentiality covers this research in order to ensure your privacy. This means that we cannot be forced to disclose any research information that may identify you, even by a court subpoena, in any federal, state, or local civil, criminal, administrative, legislative, or other proceedings.

The Certificate of Confidentiality does not prevent you or a member of your family from voluntarily releasing information about yourself or your involvement in this research. If an insurer, employer, or other person obtains your written consent to receive research information, then we will not use the Certificate to withhold that information. The Certificate cannot be used to resist a demand for information from the United States Government that is solely used for auditing or evaluation of federally funded projects. We may report to state or local officials evidence of harm or abuse to any vulnerable person, but we will not ask you any questions about such topics.

We would be more than happy to answer any questions you have about the study. Please call the University of Michigan Survey Research Center, toll free, at 1-8xx-xxx-xxxx or email the American Family Health Study at **afhs-info@umich.edu**. The AFHS website also has interesting information about the study — including about how the data will be used to inform American public health policies and programs — at http://afhs.isr.umich.edu.

If you have questions about your rights as a research participant, or wish to obtain information, ask questions or discuss any concerns about this study with someone other than the researcher(s), please contact the University of Michigan Health Sciences and Behavioral Sciences Institutional Review Board, 2800 Plymouth Road, Building 520, Room 1169 Ann Arbor, MI 48109-2800, (734) 936-0933 [or toll free, (866) 936-0933], irbhsbs@umich.edu. Please refer to protocol HUM00167171.

The Principal Investigator leading this study is Dr. Brady T. West of the University of Michigan. This study is supported by the Eunice Kennedy Shriver National Institute of Child Health & Human Development of the National Institutes of Health under Award Number R01HD095920. The content is solely the responsibility of the authors and does not necessarily represent the official views of the National Institutes of Health.

You may keep this page for your records.
