# Supplementary material for: Methods for improving participation rates in national self-administered web/mail surveys: Evidence from the United States
Source: PLoS One. 2023 Aug 4;18(8):e0289695. doi: 10.1371/journal.pone.0289695 (PMC10403122; doi:10.1371/journal.pone.0289695)
Supplement: Attachment — Submitted filename: Response to Reviewers.docx [file pone.0289695.s003.docx]

**PLOS ONE Manuscript PONE-D-22-29437R1: Responses to Reviewer Comments**

**Note:** All changes described below have been tracked in the “Revised Manuscript with Tracked Changes” file that has been submitted for this second revision. We were thankful for the opportunity to resubmit this manuscript a second time for further consideration.

**Reviewer 1**

**COMMENT 1.1:** I don't have any more comment on this manuscript. The revised version of the manuscript has come up very well. This would be a valuable knowledge addition to the science of the survey research method within and beyond USA.

**RESPONSE 1.1:** We thank Reviewer 1 for the additional review of the revised manuscript and the very positive feedback.

**Reviewer 2: No additional comments provided.**

**Reviewer 3**

**COMMENT 3.1:** I am glad to see that you have given careful consideration to all reviewer comments and in most cases have taken appropriate action that, overall, has greatly improved the paper in my view. However, one major concern remains. And I have a few additional comments related to material that you have now clarified or added.

**RESPONSE 3.1:** We are once again grateful for the additional critical review by Reviewer 3. We have addressed all remaining points below and in the revised manuscript.

**COMMENT 3.2:** My major concern is with what is now hypothesis 2. The way this hypothesis is tested does not appear to me to provide any useful information for informing future designs. This is because you test the effect of being successfully contacted. This is not a design feature that can be controlled: the survey researcher cannot choose which sample members will be successfully contacted. Rather, this is an outcome of the process. I would strongly prefer you drop this hypothesis and analysis from the paper: I think it severely weakens the paper and does not give a good impression of the authors (unlike the analyses of the other two hypotheses, which are underpinned by robust experimentation). Alternatively, you could just include a descriptive analysis (estimate) of the overall effect of the telephone reminders for the whole sample. In practice, this is the treatment that researchers can implement: they cannot control who will provide a phone number or who can be successfully contacted, so the outcome for the total sample is the metric of interest. Of course, you have no direct evidence of the counterfactual, so you can only present as a maximum bound on the effect the difference between to total observed response and the total response if you remove all who responded subsequent to a successful phone contact (true effect is probably a little smaller as some of those contacted by phone may have eventually participated anyway).

**RESPONSE 3.2:** In response to this remaining major concern, we have now deleted what was Hypothesis 2 from the paper entirely (including the subsection of the Introduction leading up to it). We have also re-labeled the third hypothesis in the first revision as the second hypothesis throughout the revised paper. The Introduction now focuses entirely on what were previously Hypotheses 1 and 3. We have included the suggested descriptive analysis in the revised subsection of the Results section focusing on response rates. Please see the new version of Table 1 for the new descriptive results, along with the new accompanying text in both the Results section and the Discussion section. To assist with interpretation of the new Table 1, we have clarified the four phases of the main stage data collection protocol in our initial description of that protocol. We have consistently emphasized all of the good points raised here by Reviewer 3 when interpreting these results, both in the revised Results section and the revised Discussion section.

**COMMENT 3.3:** p.7, l.11-12: I don’t understand this. If screener completion rate was 53.0% and weighted completion rate for main survey was 33.0%, how is the overall rate 40.7%? Surely it’s around 0.53 * 0.33 = 0.175 (if you assume screener response rate to be independent of eligibility for main stage). What am I missing?

**RESPONSE 3.3:** We again thank Reviewer 3 for the good catch here. This was in fact a typo, but we do note that this study was reporting *weighted* response rates (which prevents the simple calculation being suggested here). We removed reference to the main response rate all together, as that was not relevant to the point that we were trying to make here and just adding confusion. Our focus was on the effects of the incentives on the screening response rate. This simplifies the summary of the relevant parts of this study.

**COMMENT 3.4:** p.9, l. 5-6: I still don’t see any evidence that completion rates have benefitted from a web/mail approach? This doesn’t seem to have been compared with any other approach? Maybe you mean that they have benefitted from incentives in the context of a web/mail approach?

**RESPONSE 3.4:** Reviewer 3 is correct, and we have changed this language accordingly.

**COMMENT 3.5:** p.9, l.6: “$2 incentive was effective”: reader could be excused for assuming this means relative to no incentive. But actually the evidence you presented was that $2 was as effective as $5.

**RESPONSE 3.5:** We have further clarified this point where indicated.

**COMMENT 3.6:** p.17, l.17-19: thank you for attempting to clarify this. However, this information contradicts the notes to figure 1 (“If invited cases did not respond to mod 1, the later invitation would invite them to complete mods 1 and 2 together” and “Nonresponse to earlier modules resulted in continued invitation”). So this still requires clarification!

**RESPONSE 3.6:** We have further clarified this point where indicated (see also the footnote for the new Table 1).

**COMMENT 3.7:** p.18, l.3-9: This text is written as if it is always the same person completing screener and main questionnaire, which is presumably not the case?

**RESPONSE 3.7:** This was a typo, and should have referred to percentages of individuals from *households* completing the screening questionnaire online (since screening response was at the household level). We have revised the language here to clarify this point. Thank you again for another good catch.

**COMMENT 3.8:** p.21, l.7-11: Related to my main concern above, this adjustment – upon which your estimation relies – is likely to be woefully inadequate. I doubt these variables account for more than a small fraction of the variation in propensity to respond to a survey. So, what remains is that more willing people turn out to be more willing….

**RESPONSE 3.8:** We have deleted all references to this multivariable adjustment approach and these results from the revised manuscript, in response to your earlier major concern.
